# Coupling of equatorial Atlantic surface stratification to glacial shifts in the tropical rainbelt

**DOI:** 10.1038/s41598-017-01629-z

**Published:** 2017-05-08

**Authors:** R. C. Portilho-Ramos, C. M. Chiessi, Y. Zhang, S. Mulitza, M. Kucera, M. Siccha, M. Prange, A. Paul

**Affiliations:** 1 MARUM - Center for Marine Environmental Sciences, University of Bremen, Bremen, Germany; 2 School of Arts, Sciences and Humanities, University of São Paulo, São Paulo, Brazil; 3 Institute of Geosciences, University of São Paulo, São Paulo, Brazil

## Abstract

The modern state of the Atlantic meridional overturning circulation promotes a northerly maximum of tropical rainfall associated with the Intertropical Convergence Zone (ITCZ). For continental regions, abrupt millennial–scale meridional shifts of this rainbelt are well documented, but the behavior of its oceanic counterpart is unclear due the lack of a robust proxy and high temporal resolution records. Here we show that the Atlantic ITCZ leaves a distinct signature in planktonic foraminifera assemblages. We applied this proxy to investigate the history of the Atlantic ITCZ for the last 30,000 years based on two high temporal resolution records from the western Atlantic Ocean. Our reconstruction indicates that the shallowest mixed layer associated with the Atlantic ITCZ unambiguously shifted meridionally in response to changes in the strength of the Atlantic meridional overturning with a southward displacement during Heinrich Stadials 2–1 and the Younger Dryas. We conclude that the Atlantic ITCZ was located at ca. 1°S (ca. 5° to the south of its modern annual mean position) during Heinrich Stadial 1. This supports a previous hypothesis, which postulates a southern hemisphere position of the oceanic ITCZ during climatic states with substantially reduced or absent cross-equatorial oceanic meridional heat transport.

## Introduction

**Figure 1:**
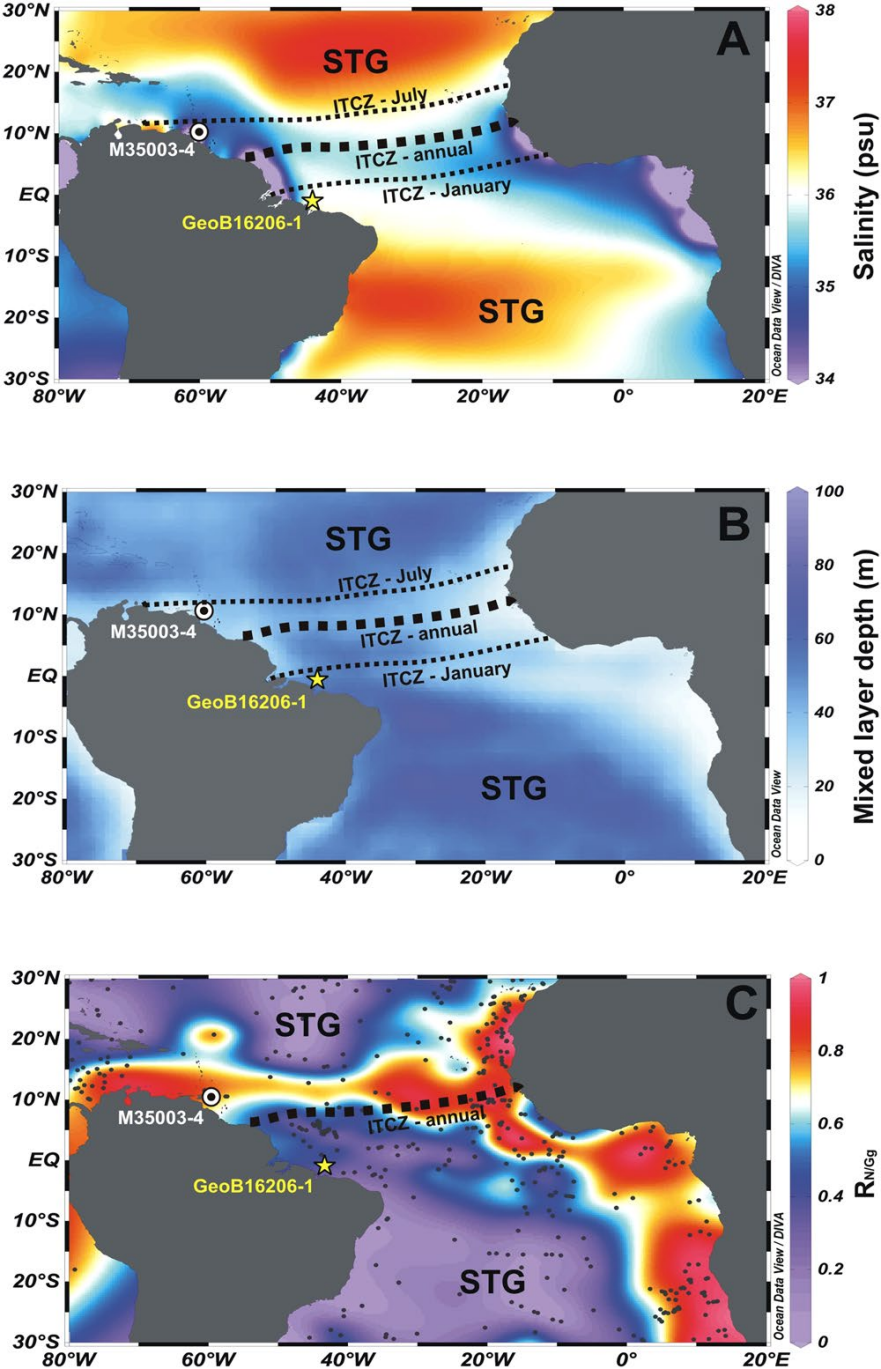
Tropical Atlantic Ocean maps with location of the investigated cores, upper water column properties and modern planktonic foraminifera response to the mean annual mixed layer depth. (**A**) Mean annual surface salinity (WOA 2009^29^) and location of cores GeoB16206–1 (1°34.75′S, 43°01.42′W) and M35003–4 (12°5.4′N/61°14.6′W). The black dashed lines indicate the mean annual (thick) and seasonal (thin) position of the Intertropical Convergence Zone (ITCZ). The central portions of the subtropical gyres (STG) are indicated. (**B**) Mean annual mixed layer depth^6, 7, 49^. (**C**) Distribution of the R_N/Gg_ ratio. Black dots represent the location of the 407 surface sediment samples^28^. Figure created using Ocean Data View software^50^ (ODV - version, 4.7.4., http://odv.awi.de, 2015).

The Intertropical Convergence Zone (ITCZ) can be defined as a narrow belt of maximum tropical precipitation associated with the ascending branch of the Hadley circulation^1^. It is one of the most prominent features of the general atmospheric circulation, regulating the tropical hydrologic cycle and the cross–equatorial atmospheric energy transport^2, 3^. The position of the ITCZ is linked to meridional sea surface temperature (SST) gradients, resulting in predictable latitudinal shifts in line with the seasonal cycle^3, 4^. Primarily because of the meridional ocean–atmospheric energy transport, the annual average position of the ITCZ is not located at the equator, but north of it (Fig. 1a)^1, 3, 5^. At the tropical Atlantic surface, the position of the ITCZ is marked not only by a low-salinity belt (Fig. 1a) but also by a prominent change in water column structure. Excess of freshwater flux below the ITCZ changes the density of the upper water column by creating a thin low salinity surface layer which hampers efficient wind-driven vertical mixing^6, 7^ and results in a pronounced shallow mixed layer between 5°N–12°N (Fig. 1b; Supplementary Information and Supplementary Fig. S2). This upper ocean feature is considered to be the oceanic counterpart of the ITCZ. The shallow mixed layer allows the presence of cold and nutrient–rich thermocline waters in the photic zone (Supplementary Fig. S2), steepening the thermal vertical gradient and boosting regional primary productivity^8^. Indeed, the resulting shallow tropical mixed layer together with the elevated productivity is recorded in the composition of planktonic foraminifera faunas in the seafloor sediments below, which could be used to track the mean position of the oceanic counterpart of the ITCZ in the equatorial Atlantic (i.e., the Atlantic ITCZ).

Paleoceanographic records suggest recurrent millennial-scale events of disrupted cross-equatorial heat transport due to slowdowns of the Atlantic Meridional Overturning Circulation (AMOC)^9, 10^, associated with massive fluxes of icebergs and melt water into the North Atlantic^11^ (i.e., Heinrich Stadials, HS). As a result, the ITCZ adjusted its position, reflecting the change in interhemispheric heat transport, migrating towards the warmer hemisphere, in this case, southward^11^. While the changes in the position of the ITCZ over land are increasingly well documented due to the associated shifts in rainfall patterns^12–16^, it remains unclear whether and to which extent the Atlantic ITCZ also changed in response to millennial-scale global climate forcing^17, 18^. Since the tropical sea surface temperature (SST) gradient modulates the meridional migration of maximum tropical precipitation associated to the ITCZ^4^, some studies have reconstructed the tropical SST gradient as well as sea surface salinity changes in order to estimate the past position of the Atlantic ITCZ^17, 18^. However, the temporal resolution of the available records does not allow the evaluation of the behavior of the Atlantic ITCZ at millennial-scale. Thus, new proxies and high-temporal resolution records that adequately track the mean position of the Atlantic ITCZ during millennial-scale events are required.

Here we provide a new proxy for the mixed layer depth based on the relative abundance of three planktonic foraminifera species and apply it to track the position of the Atlantic ITCZ during the last 30 cal ka BP in high-temporal resolution. We compared the modern distribution of planktonic foraminifera in surface sediments from the Atlantic Ocean between 30°N and 30°S with the water mass distribution to identify a suitable group of species that appropriately respond to the shallowest mixed layer associated with the Atlantic ITCZ (see Materials and Methods and Supplementary information). As a result, *Neogloboquadrina dutertrei, Neogloboquadrina incompta* and *Globigerina glutinata* were identified as key species to track the modern position of the Atlantic ITCZ in space. The east–west belt of higher abundance of *N. dutertrei* and *N. incompta*, accompanied by minimum abundance of *G. glutinata* between 5° and 15°N reflects to the mean annual position of the ITCZ (5°–12°N) (Supplementary Information and Fig. S1). Considering the opposing abundance patters of both *Neogloboquadrina* species and *G. glutinata* in the equatorial Atlantic Ocean, we propose that the ratio R_N/Gg_ = %*Neogloboquadrina*/(%*Neogloboquadrina* + %*G. glutinata*) can be used as a proxy for the modern position of the shallowest mixed layer associated with the Atlantic ITCZ (Fig. 1c). These species as well as the R_N/Gg_ ratio respond well to the significant changes in the hydrographic and trophic structure of the upper ocean associated with the shallowest mixed layer related to the ITCZ (Supplementary Fig. S2).

To reconstruct the variability of the Atlantic ITCZ over time, we applied the R_N/Gg_ ratio to two sediment cores located on opposite sides of the modern seasonal range of the Atlantic ITCZ (Fig. 1a), synchronized based on high–resolution radiocarbon chronologies (Supplementary Table 1). The position of the cores has been carefully selected such that a meridional shift of the Atlantic ITCZ should cause an antiphased behavior of the R_N/Gg_ records of both cores. Core GeoB16206–1 (1°34.75′S/43°01.42′W/1367 m water depth) was raised off NE Brazil (Fig. 1) and features exceptionally high sedimentation rates during HS1 (up to 93 cm kyr^–1^) and the YD (~60 cm kyr^–1^) (Supplementary Fig. S3)^13^. Core M35003–4 (12°5.4′N/61°14.6′W/1300 m water depth) was raised from the Tobago Basin^19, 20^ (Fig. 1) and its AMS ^14^C ages were recalibrated to make both records comparable (see Materials and Methods). The planktonic foraminifera assemblage was newly determined in GeoB16206–1 while that from core M35003–4 was taken from Hüls and Zahn (ref. 20) (Supplementary Fig. S6). The subsurface temperatures were reconstructed at both sites using a planktonic foraminifera–based Modern Analogue Technique (MAT) (see Materials and Methods). We also analyzed the Ti/Ca ratio in GeoB16206–1 to infer millennial-scale increases in river runoff associated with pulses of ITCZ-related rainfall over NE Brazil^12–14^. Additionally, we used the output from a transient simulation of the last deglaciation (provided by the TraCE-21ka project, http://www.cgd.ucar.edu/ccr/TraCE/)^21^ using a comprehensive coupled atmosphere-ocean general circulation model^22^ to assess the effects of the AMOC slowdown during HS1 on the meridional displacement of the ITCZ and on the mixed layer depth of the tropical Atlantic (see Materials and Methods for a detailed design of model simulations).

**Figure 2:**
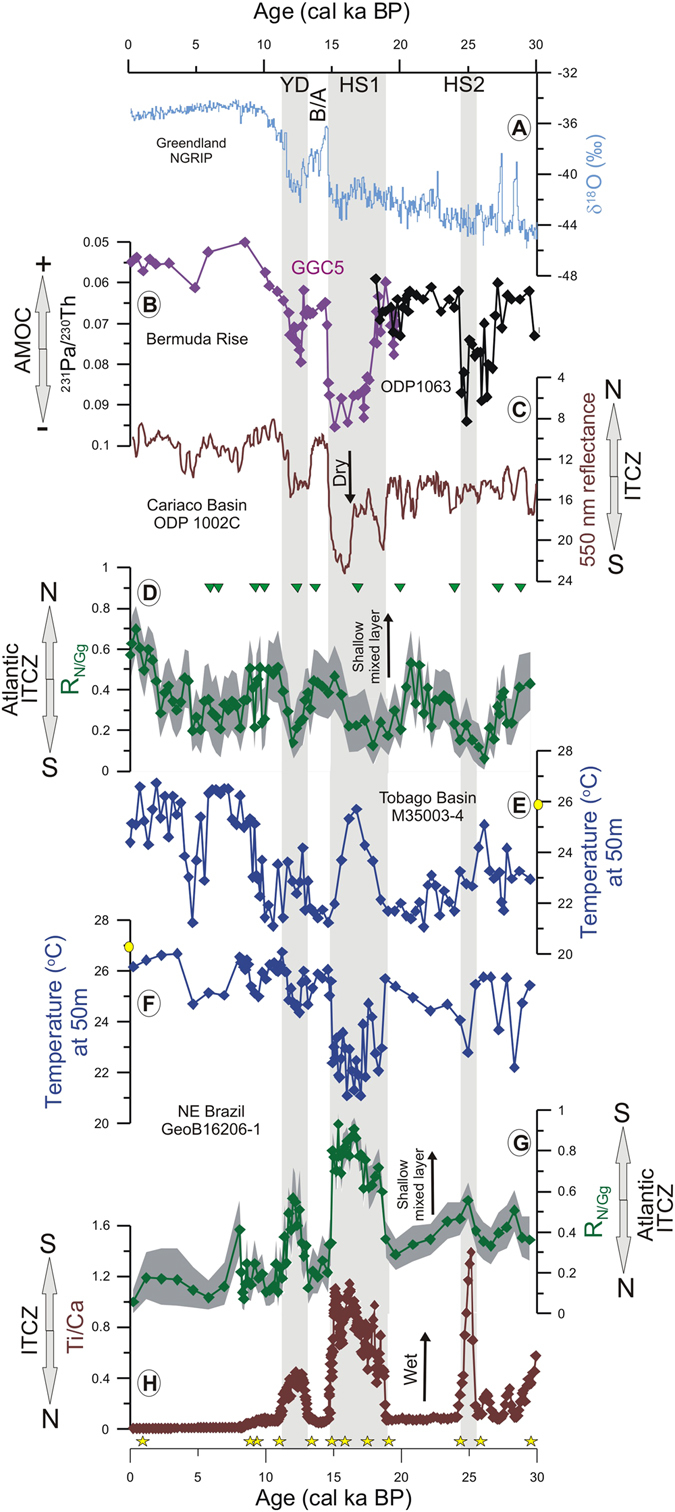
Antiphase relationship of the upper water column off NE-Brazil and at the Tobago Basin over the last 30 cal ka BP. (**A**) Greenland δ^18^O record^51^; (**B**) Bermuda Rise cores GGC5 (purple) and ODP1063 (black) ^231^Pa/^230^Th as a proxy for the strength of the Atlantic Meridional Overturning Circulation (AMOC)^9, 10^; (**C**) Cariaco Basin core ODP1002C reflectance showing wet/dry conditions linked to meridional shifts of the Intertropical Convergence Zone (ITCZ)^15^; %*Neogloboquadrina*/(%*Neogloboquadrina* + %*G. glutinata*) (R_N/Gg_) ratio as a proxy for the position of the Atlantic ITCZ in the (**D**) Tobago Basin core M35003–4 and in the (**G**) NE Brazil core GeoB16206–1; Modern Analog Technique-based temperature at 50 m water depth in the (**E**) Tobago Basin and (**F)** off NE Brazil; (**H**) Ti/Ca ratio as a proxy for precipitation over NE Brazil. Grey shading in (**D**) and (**G**) indicates the 95% confidence interval. Vertical gray bars indicate Heinrich Stadials 2 and 1 (HS2–1) and the Younger Dryas (YD). The Bølling–Allerød (B/A) is also indicated. Calibrated AMS ^14^C ages are shown as green triangles for core M35003–4^19, 20^ and yellow stars for core GeoB 16206–1^3^. Yellow circle in (**E**) and (**F**) indicate modern temperature at core locations^29^. Note the inverted axes in (**B**) and (**C**).

Off NE Brazil, the abundances of *N. dutertrei* and *N. incompta* show an opposite pattern to that of *G. glutinata* over the last 30 cal ka BP (Supplementary Fig. S5a–d). High R_N/Gg_ values (≥0.5) are accompanied by a strong cooling of subsurface waters (2–4 °C) during HS2–1 and the YD (Fig. 2f,g, Supplementary Fig. S5d,e). Changes in the R_N/Gg_ ratio and subsurface temperatures are synchronous to changes in ^231^Pa/^230^Th from the Bermuda Rise^9, 10^ and suggest a fast response of the assemblages of planktonic foraminifera off NE Brazil to changes in the strength of the AMOC (Fig. 2b,f,g). This fast response is consistent with the development of a rainfall-induced shallow mixed layer off NE Brazil caused by the a southward migration of the ITCZ during the HS2–1 and the YD^12–14, 23^. Indeed, higher R_N/Gg_ values as well as cooler subsurface waters occurred simultaneously with increased ratios of Ti/Ca from core GeoB16206–1 during HS2–1 and the YD (Fig. 2f,h), highlighting the tight connection between the position of the shallowest mixed layer and the position of the tropical rainbelt by the meridional movements of the ITCZ. The subsurface cooling can partly be attributed to a reduced downward mixing of heat from the strongly salinity-stratified surface layers^6–8^.

A clear antiphase relationship is observed in both the R_N/Gg_ and subsurface temperature records off NE Brazil and the Tobago Basin (Fig. 2d–g, Supplementary Fig. S7b–e). Elevated values of R_N/Gg_ and cooler subsurface waters off NE Brazil occurred simultaneously (i.e., within age model uncertainties) with decreased R_N/Gg_ values (deeper mixed layer) and warmer subsurface waters (partly due to enhanced turbulent downward mixing of heat) in the Tobago Basin during HS2–1 and the YD. The deglacial antiphase behavior is supported by the significant anticorrelation of R_N/Gg_ (Pearson correlation coefficient r is −0.50 with 95% confidence interval [−0.72; −0.19], see Materials and Methods) and subsurface temperatures at 50 m (r = −0.72 with 95% confidence interval [−0.87; −0.46]) between the records off NE Brazil and the Tobago Basin. This antiphasing strongly suggests that the meridional movements of the Atlantic ITCZ influenced both sites during millennial–scale climate oscillations with a southward shift during HS2–1 and the YD (Fig. 2). At the northern range of the modern ITCZ seasonal migration, the high–temporal reflectance record from Cariaco Basin indicates dry conditions near the Tobago Basin off northern South America during HS1 and the YD due to the southward displacement of the ITCZ^15^ (Fig. 2c). On the opposite side of the seasonal migration range of the ITCZ, our Ti/Ca record (Fig. 2h) is consistent with geochemical proxies^12–14^, speleothems^14, 24^, model simulations^23^ and palynological information^25^ from continental and marine archives collected around NE Brazil that indicate increased rainfall. Taken together, these records strongly indicate that the ocean–atmosphere systems associated with the ITCZ shifted meridionally during millennial–scale climatic oscillations of the last 30 cal ka BP.

We assume the influence of the Parnaiba River runoff on the GeoB16206–1 R_N/Gg_ record to be negligible. Our assumption is supported by the fact that, in the Amazon River plume, R_N/Gg_ displays intermediate values (~0.4–0.5) probably associated with a large freshwater flux, which leads to a relatively shallow mixed layer^6, 26^ (Fig. 1c and S1f). Since the Parnaíba River discharge is several orders of magnitude lower than that of the Amazon discharge, higher values of R_N/Gg_ (≥0.6) found off NE Brazil during the HS2–1 and the YD are unlikely to be explained solely by increased Parnaíba River runoff. We argue that the highest values of R_N/Gg_ recorded in core GeoB16206–1 were primarily caused by the southward shift and prolonged presence of the Atlantic ITCZ off NE Brazil during Heinrich-like events.

**Figure 3:**
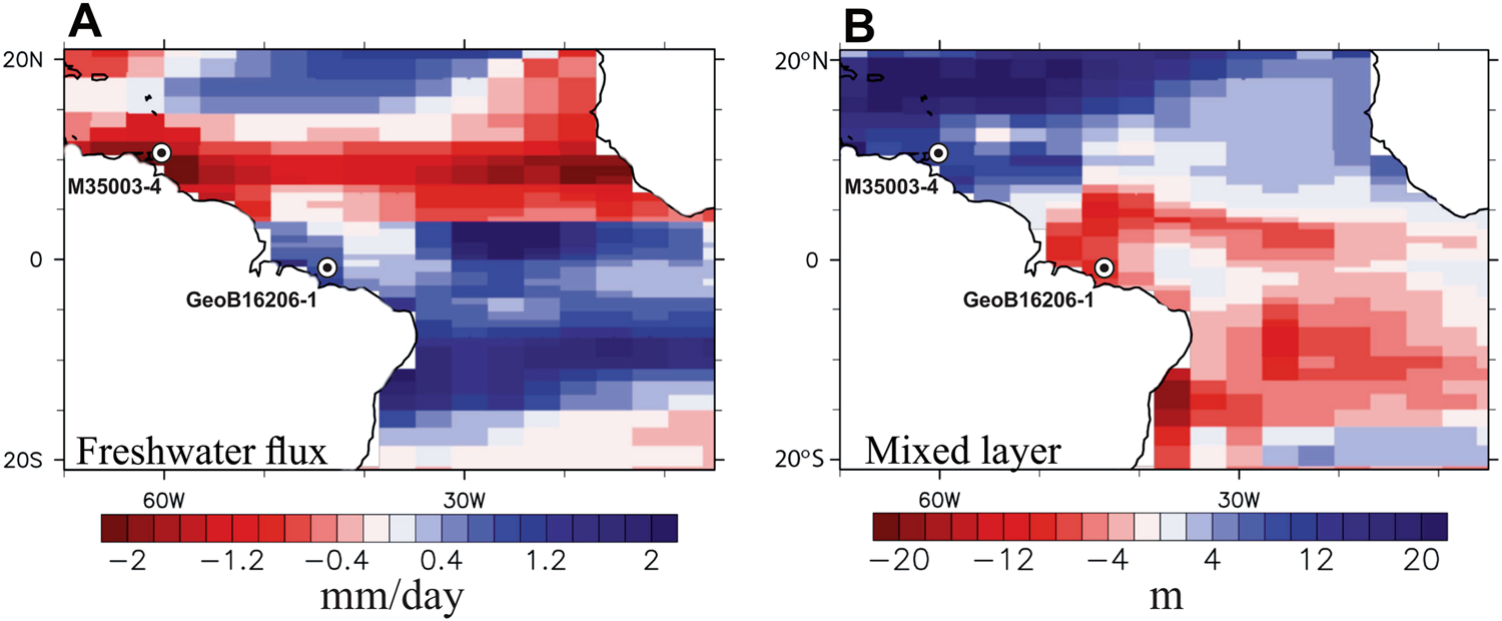
Simulated response of tropical Atlantic Ocean surface freshwater flux and mixed layer depth to a slowdown of the Atlantic Meridional Overturning Circulation. Shown are the long–term mean (**A**) surface freshwater flux (precipitation plus continental runoff minus evaporation) and (**B**) mixed layer depth anomalies during Heinrich Stadial 1 (18–15 ka average) relative to the Last Glacial Maximum (LGM) (22–19 ka average) from the transient TraCE-21ka deglacial simulation^22^. Starting from the LGM, the coupled climate model (CCSM3) was integrated through the last deglaciation, subject to varying forcing by orbital insolation, atmospheric greenhouse gas concentrations, continental ice sheets, and meltwater fluxes. Figure created using NCL (NCAR Command Language, www.ncl.ucar.edu)^52^.

The TraCE-21k coupled atmosphere-ocean transient simulation of the last deglaciation also shows a clear antiphase pattern between the region off NE Brazil and the Tobago Basin for both the mixed layer depth and the annual mean surface ocean freshwater flux in response to a slowdown of the AMOC (Fig. 3). During HS1, the simulation shows a positive anomaly (relative to the LGM) in net precipitation off NE Brazil and a negative anomaly over the Tobago Basin owing to a southward shift of the ITCZ (Fig. 3a). These results corroborate previous model simulations^4^. Importantly, in agreement with our R_N/Gg_ and subsurface temperature records (Fig. 2d–g), the ITCZ shift goes along with a change in tropical Atlantic mixed layer depths (Fig. 3b). The model results therefore strongly support the notion of an ITCZ-driven shift in mixed layer depth, although the effects of anomalous surface freshwater fluxes on tropical mixed layer depth may be modified by wind-stress and circulation anomalies.

Our results indicate that the upper water column off NE Brazil responded to a positive anomaly of freshwater flux promoted by a southward shift of the ITCZ through a shoaling of the mixed layer and changes in the plankton community during HS2–1 and the YD. Physical and ecological changes in the upper tropical Atlantic followed southward displacements of the Atlantic ITCZ triggered by decreases in cross-equatorial heat transport associated with slowdowns in the AMOC. The investigated records indicate that, in the western tropical Atlantic, the Atlantic ITCZ may have been located south of the equator at ca. 1°S (with a mean displacement of ca. 5° from its modern mean annual position) during Heinrich Stadial 1, findings that are supported by climate model simulations. Therefore, our results support the model-derived hypothesis of an ITCZ position to the south of the equator under a markedly reduced cross-equatorial oceanic heat transport^1^.

## Materials and Methods

### Modern planktonic foraminifera and oceanic properties

The 407 surface sediments samples from the Atlantic Ocean used to characterize our proxy for the mixed layer depth span 30°N – 30°S and have been summarized by the MARGO Project^27, 28^ (Figs S1c–f, S2a,f). The abundances of planktonic foraminiferal species in these samples were compared with mean annual physico–chemical properties (i.e., temperature, salinity, oxygen and phosphate concentration) from the World Ocean Atlas 2009 (WOA 2009^29–32^). All foraminiferal samples were picked from >150 µm size fraction of sample splits containing approximately 300 specimens^27, 28^. The ages of the majority surface sediments (392 samples) fall into chronostratigraphic levels 4 and 5 as defined by the MARGO Project^27, 28^. Level 4 classifies surface samples with an age range of 0–4 cal ka BP where the chronologic control is based on stratigraphic constraints such as δ^18^O and %CaCO_3_. Level 5, in turn, describes surface samples that have no age control^27^. The foraminiferal data used here are available from the World Data Center PANGAEA (https://doi.pangaea.de/10.1594/PANGAEA.841194).

### Marine sediment cores

To reconstruct the variability of the Atlantic ITCZ over time, we used two high- resolution marine sediment cores from the western tropical Atlantic Ocean, located on opposite sides of the modern ITCZ (Fig. S1). Core GeoB16206–1 (1°34.75′S, 43°01.42′W, 1367 m water depth) was raised off NE Brazil^33^ close to the southernmost position (January) of the modern annual migration cycle of the atmospheric ITCZ. Core M35003–4 (12°5.4′N/61°14.6′W/1300 m water depth) was raised from the Tobago Basin below the northernmost position of the ITCZ (July) (Fig. S1b). Core GeoB16206–1 is composed of weakly bioturbated to bioturbated foraminifera bearing clays, and was sampled (10 cm^3^ per sample) continuously every 10 cm (i.e., 81 samples). All samples were freeze–dried and washed through a sieve of 150 µm mesh size. Details about the lithology and sampling strategy and preparation of samples for core M35003–4 are available in Rühlemann *et al*. (ref. 19) and Hüls and Zahn (ref. 20).

### Planktonic foraminifera assemblage

All samples from core GeoB16206–1 were dry picked from the >150 µm size fraction and quantified in relative abundances from splits containing more than 300 specimens per sample. The taxonomy was based on Stainforth *et al*.^34^ and Hemleben *et al*.^35^. The species *Neogloboquadrina dutertrei* was distinguished from *Neogloboquadrina pachyderma* (d) (=*Neogloboquadrina incompta*) by the occurrence of an umbilical tooth and the presence of more than four chambers in N. dutertrei^36^. We assumed the effect of dissolution in our planktonic foraminiferal faunal composition to be negligible since core Geob16206–1 was collected at 1367 m water depth, well above the modern and glacial lysocline^37^. Planktonic foraminifera faunal composition data for core M35003–4 was previously published by Hüls and Zahn (ref. 20) (Fig. S6). Here we propose the use of the ratio R_N/Gg_ = %*Neogloboquadrina*/(%*Neogloboquadrina* + %*G. glutinata*) as a proxy for mixed layer depth, which can be used to track the modern position of the Atlantic ITCZ. The confidence intervals of the R_N/Gg_ ratio (95%) were determined by random subsampling (1000 bootstrap cycles) of 300 individuals of a modeled planktonic foraminifera assemblage (N = 10000) with given relative abundances of the *G. glutinata* and both *Neogloboquadrina* species. The lower (2.5%) and upper (97.5%) boundaries of the respective confidence intervals are shown in Fig. 2d,g.

### Age model

The chronology of sediment core GeoB16206–1 has recently been published in Zhang *et al*.^13^ and is based on 12 AMS radiocarbon measurements of the planktonic foraminifer *Globigerinoides sacculifer* performed at the Poznan Radiocarbon Laboratory in Poland (Table S1). Raw ages were calibrated using the Calib 7.0 software^38^ and the Marine13 radiocarbon calibration curve^39^. Ages between calibrated ^14^C AMS values were linearly interpolated, and expressed in calibrated kiloannum B.P. (cal ka BP) (Fig. S3). To make both records readily comparable, we recalibrated the AMS ^14^C ages from core M35003–4^19, 20^ using the same procedure described above.

### Subsurface temperature reconstruction

Subsurface temperatures from sites GeoB16206–1 and M35003–4 were estimated from a planktonic foraminifera transfer function determined by the Modern Analogue Technique (MAT) using the software C2^40^. The MAT compares fossil samples with a given calibration dataset and selects those with the most similar faunal composition as analogs for environmental conditions. The planktonic foraminifera calibration dataset used here comprised the previously mentioned 407 surface samples from the Atlantic Ocean between 30°N and 30°S from the MARGO database^27, 28^. Temperature reconstructions derived from transfer functions based on planktonic foraminifera are usually calibrated against fixed near–surface (i.e., 10 m) values^41^. However, Telford *et al*.^41^ demonstrated that planktonic foraminifera are more sensitive to subsurface than surface temperatures. Hence, temperature reconstructions based on a fixed near–surface water depth (e.g., 10 m water depth) can be biased. Since more than 70% of the planktonic foraminifera species present in both cores (i.e., *Globigerinoides ruber*, *G. sacculifer, G. glutinata* and *Globigerinella siphonifera*) inhabit the upper 60 m of the water column^42–44^, we extracted and calibrated the modern annual temperature values for 50 m water depth from WOA 2009^29^. This also allowed us to assess subsurface temperatures directly affected by the Atlantic ITCZ (Fig. S2b). For our MAT transfer function we employed the squared chord distance as a similarity measure und used the weighted mean of the best 10 modern analogues as reconstruction result^27^. Using the leave–one–out cross–validation method, the root mean square error of prediction (RMSEP) of the transfer function was calculated as 1.02 °C (R^2^ = 0.90) (Fig. S4).

### Major element composition

The intensities of major elements of sediment core GeoB16206–1 were determined with the X–ray fluorescence (XRF) core–scanner II (AVAATECH Serial No.2) at MARUM, University of Bremen. Scanning was performed directly over the split core surface of the archive half. Analyses were performed every 2 cm step over a 0.15 cm^2^ area for 20 seconds with current of 10 kV. Besides, we also measured major element concentrations of 37 bulk sediment samples to calibrate scanner intensities. Samples of about 10 ml (~5 g dry sediment) of core GeoB16206–1 were freeze–dried, powdered and homogenized, and then prepared for measurement by energy dispersive polarization X–ray fluorescence (EDP–XRF) spectroscopy at MARUM. We applied a log–ratio regression approach^45^ to calibrate the proportions of six major elements (i.e., Ca, Fe, Al, Si, Ti and K). Here we show the ratio between calibrated proportions of Ti and Ca.

### Coupled atmosphere-ocean TraCE-21ka simulation

To support our proxy-derived finding of ITCZ-induced shifts of the tropical Atlantic mixed layer, we analyzed the output from the transient TraCE-21ka simulation of the last deglaciation^22^. This simulation uses the Community Climate System Model version 3 (CCSM3) of the National Center for Atmospheric Research, which is composed of four separate components representing the atmosphere, ocean, land, and sea ice^46^. The resolution of the atmosphere component is T31 (correspond to a 3.75° transform grid) in the atmosphere, with 26 layers in the vertical, while the ocean and sea-ice components have a nominal resolution of 3° with refined meridional resolution (up to 0.9°) around the equator and 25 levels in the vertical^47^. Starting from the Last Glacial Maximum (LGM), the model was integrated through the last deglaciation subject to changes in insolation, atmospheric greenhouse gas concentrations, continental ice sheets, and meltwater fluxes^21^. Owing to the anomalous North Atlantic meltwater input, the model simulates a decrease of the AMOC during Heinrich Stadial 1 (HS1) from ca. 13 Sv during the LGM to about 4 Sv during the HS1^22^. For our analysis of HS1 tropical Atlantic surface freshwater flux and mixed layer depth anomalies, we averaged the output fields from 18–15 ka and compared them to the mean LGM state (22–19 ka average).

### Correlation analyses

Pearson correlation coefficients between proxy time series and the corresponding 95% confidence intervals were calculated using the PearsonT3 software (http://www.manfredmudelsee.com/soft/pearsont/index.htm). The software estimates the correlation coefficient (r) with accurate bootstrap confidence intervals by accounting for the autocorrelation of the data^48^. Data sets were re-sampled for the interval 20–10 ka BP (glacial termination which includes Heinrich Stadial 1 and the Younger Dryas) at 0.1-kyr steps on the basis of their original age models to achieve the same timescale.

Full Methods and any associated references are available in the online version of the paper at www.nature.com/nature.

## Electronic supplementary material

Supplementary information

Table S1
