# Supplementary material for: Coupling of equatorial Atlantic surface stratification to glacial shifts in the tropical rainbelt
Source: Sci Rep. 2017 May 8;7:1561. doi: 10.1038/s41598-017-01629-z (PMC5431516; doi:10.1038/s41598-017-01629-z)
Supplement: Supplementary file 1 — Supplementary information [file 41598_2017_1629_MOESM1_ESM.doc]

**Supplementary Information**

**Coupling of equatorial Atlantic surface stratification to glacial shifts in the tropical rainbelt**

**Portilho-Ramos, R.C.^1,*^; Chiessi, C.M.^2^; Zhang, Y.^1^; Mulitza, S.^1^; Kucera, M.^1^; Siccha, M.^1^; Prange, M.^1^, Paul, A.^1^**

1-MARUM - Center for Marine Environmental Sciences, University of Bremen, Bremen, Germany

2-School of Arts, Sciences and Humanities, University of São Paulo, São Paulo, Brazil

*-Presently at the Institute of Geosciences, University of São Paulo, São Paulo, Brazil

**Supplementary Regional Setting**

The continental margin off NE Brazil is under the influence of the warm, salty and oligotrophic waters of the North Brazil Current (NBC) (Fig. S1a). The NBC originates around 15°S after the South Equatorial Current (SEC) splits into the southward flowing Brazil Current (BC) and the northward flowing NBC^1,2^  (Fig. S1a). The NBC is a crucial component of the cross–equatorial northward transfer of heat and salt through surface waters that compensate for the formation and southward flow of North Atlantic Deep Water (NADW)^1–3^. It is estimated that 15**–**16 Sv from the total transport of the NBC is associated with NADW formation in high northern latitudes^4,5^. During austral winter and spring (June to November), the Intertropical Convergence Zone (ITCZ) is at its northernmost position (~12°N) (Fig. S1b)^6,7^, the SE trade–winds are stronger, the NBC transport is intense and the thermocline is deeper (shallower) in the western (eastern) equatorial Atlantic^4,8^. In contrast, during austral summer and fall (December to May), the ITCZ reaches its southernmost position (~5°N) (Fig. S1b)^6,9^ , the NE trade–winds are stronger, the NBC is weak, the zonal pressure gradient relaxes and the thermocline in the western equatorial Atlantic shoals^4,8^. Meridional movements of the ITCZ and of the intensity of the easterly trade–winds are responsible for an outstanding feature in the upper equatorial Atlantic, the Atlantic Equatorial Divergence Zone (AEDZ)^3,9,10^ (Fig. S2b–e).

The AEDZ is of crucial importance to the ocean–atmosphere system and the Atlantic meridional heat budget constituting a major tropical upwelling zone of thermocline waters between 10°N**–**10°S promoted by trade–wind stress and Ekman transport^3,9^ (Fig. S2b–e). With an Ekman divergence of 23–25 Sv^10,11^ surface waters are transported towards the poles allowing the subducted water from the subtropical gyres to upwell in the equatorial region as part of the shallow meridional overturning referred to as subtropical cells (STC)^3,10^. It is estimated that ~6 Sv of the thermocline waters that upwells in the tropical Atlantic flows toward the North Atlantic (through the NBC) to compensate for the formation of North Atlantic Deep Water as a part of the warm return flow of the thermohaline circulation^3^. The transport allows permanent thermocline waters to occupy the photic zone, steepening the thermal vertical gradient and boosting biological productivity^12^. This is manifested as a tongue of cold (i.e., <18°C)^13^, low salinity (i.e., 35–35.75 psu)^14^, high nutrient (e.g., phosphate concentration of 0.75–2 µmol/l)^15^, and poorly ventilated (i.e., oxygen concentration of <4 µmol/l)^16^ waters between 10°N and 10°S (Fig. S2b–e) in the upper water column. However, a zone of thin mixed layer and shallow thermocline is observed between 5°N–12°N and coincides with the annual mean position of the ITCZ over the equatorial Atlantic (Figs. S1b and S2b-e). Intense freshwater flux below the ITCZ changes the density of the upper water column, creating a layer between the base of the mixed layer and the top of the thermocline, the so-called Barrier Layer^17–19^. Furthermore, the surface low-salinity layer below the ITCZ forms a halocline that suppresses wind-driven turbulent mixing of the upper water column^18^, allowing the thermocline to be located at its shallowest position within the AEDZ (Fig. S2b-e). Since the ITCZ and its rainbelt swing seasonally in a narrow meridional range north of the equator, a pronounced zone of shallowest mixed layer and thermocline occurs between 5°N–12°N and can be considered the ITCZ's oceanic counterpart, the Atlantic ITCZ (Figs. S1 and S2).

**Supplementary Discussion**

***Tracking the modern Atlantic ITCZ by*** p***lanktonic foraminifera***

The modern distributions of *Neogloboquadrina dutertrei* and *Neogloboquadrina incompta* show a similar pattern, being absent or rare (≤ 5%) in sediments below both subtropical gyres and off northeastern South America, but abundant (≥20%) in the eastern Atlantic Ocean (Fig. S1c–d). Interestingly, both species show an east–west belt of relatively high abundance (i.e. 5–10%) in the equatorial Atlantic between 5° and 15°N. In contrast, *Globigerina glutinata* shows an opposite abundance pattern, being abundant (i.e., ~10%) below both subtropical gyres, and off northeastern South America, and virtually absent off western Africa (Fig. S1e). In addition, *G. glutinata* abundances show an east–west belt of relative low abundances (< 5%) between 0°–15°N coinciding with the belt of higher abundances of *N. dutertrei* and *N. incompta*.

The distributions of these three species show patterns that are apparently linked to the shallow mixed layer and wind driven upwelling zones (Fig. S1a–e). Notably, the east–west belt of higher abundance of *N. dutertrei* and *N. incompta* together with the lower abundance of *G. glutinata* between 5°–15°N matches well with the annual mean position occupied by the Atlantic ITCZ (5°–12°N)^3,6,7^ (Fig. S1b–e). The small offset between species abundance and the annual mean position of the ITCZ is discussed below. *Neogloboquadrina dutertrei* and *G. glutinata* calcify at different depths in the water column^26,27^. While *N. dutertrei* calcifies close to the thermocline depth (between 90 and 135 m^26,27^), the *G. glutinata* calcifies in the upper 40m^25^. Both species appear to be associated with high productivity in warm, tropical waters^24,28^, but *N. dutertrei* seems to be linked to the depth of the chlorophyll maximum^24,29,30^ and, because of its facultative symbionts^31,32^, it can only benefit from it where the chlorophyll maximum occurs in the top 100 m^27,31^. This explains the zonation in the tropical Atlantic (Fig. S1c-e): where the mixed layer is thin (and hence the thermocline allowed to be shallow), *N. dutertrei* is abundant, where not, *G. glutinata* is. *Neogloboquadrina incompta* is a cold–subpolar to transitional species typically found in the Southern Ocean associated with the Antarctic Circumpolar Current^33^. Its higher abundance in tropical waters is closely associated with high productivity waters of the eastern Atlantic upwelling^28,22^. Thus cold subsurface waters (steeper vertical thermal gradient) and increased food availability associated with shallow mixed layer zones explains the similar distribution of *N. dutertrei* and *N. incompta* in the Atlantic Ocean (Fig. S1c,d). Due to the similar geographic distribution of *N. dutertrei* and *N. incompta* and assuming similar mechanisms controlling their distributions in the tropical Atlantic Ocean, we pooled both species under the term “*Neogloboquadrina”.*

The opposite abundance pattern of *Neogloboquadrina* and *G. glutinata* is also clear in a latitudinal cross–section in the western Atlantic from 30°N to 30°S (Fig. S2f). The latitudinal cross–section was located in order to (i) include a large number of samples from the western Atlantic, and (ii) avoid the middle–eastern Atlantic samples associated with the upwelling zones off western Africa. Both *N. dutertrei* and *N. incompta* seem to respond positively to ITCZ–triggered mixed layer shoaling, showing increased abundance within this region in comparison to both subtropical gyres where surface waters are subducted to complete the STC^3,10^ (Figs. S1c-d and S2b-e). At the center of the subtropical gyres, the deep thermocline, elevated salinity, well-ventilated and oligotrophic waters are unfavorable to *N. dutertrei* and *N. incompta* (Figs. S1c,d and S2f). Maximum abundance of both species, accompanied by minimum abundance of *G. glutinata*, coincide with the shallowest mixed layer associated with annual maximum freshwater flux (associated to the ITCZ) occurring between 5°–15°N, supporting that *N. dutertrei, N. incompta* and *G. glutinata* are key–species to trace the modern position of the Atlantic ITCZ (Figs. S1 and S2). Therefore we propose the use of the ratio between the abundance of these species as a proxy for mixed layer depth, which can be used to track the modern position of the Atlantic ITCZ (Equation 1).

1. R_N/Gg_ = %*Neogloboquadrina* / (%*Neogloboquadrina* + %*G. glutinata*)

As shown in Figures S1f and S2f, the highest values of R_N/Gg_ (~0.8) closely match the latitudes of the shallowest mixed layer between 30°N and 30°S associated to the modern mean position of the Atlantic ITCZ, supporting the applicability of the R_N/Gg_ for reconstructing its past shifts. Higher values of R_N/Gg_ (~0.8) are found in the Tobago Basin core M35003-4 associated with the modern ITCZ, whereas off NE Brazil the values of R_N/Gg_ are close to zero due to the modern deeper mixed layer. The confidence intervals of the R_N/Gg_ ratio were determined by random subsampling (1000 bootstrap cycles) of 300 individuals of a modelled planktic foraminifera assemblage (N=10000) with given relative abundances of the *G. glutinata* and both *Neogloboquadrina* species. The lower (2.5%) and upper (97.5%) boundaries of the respective confidence intervals are shown in Figures S5d, S6d and S7b, e.

***The offset between species abundance in surface sediment and the mean position of the modern ITCZ***

The east–west belt of higher abundance of *N. dutertrei* and *N. incompta*, together with the lower abundance of *G. glutinata* between 5°–15°N matches the annual mean position occupied by the Atlantic ITCZ (5°–12°N)^3,6,7^ (Fig. S1a–d). However, there is a small offset between the east–west belt of species abundance (including the maximum R_N/Gg_) and the annual mean position of the ITCZ that is likely due to slight differences between the period recorded by surface samples from MARGO^21^ and the modern database from WOA 2009^13–16^. The ages of these surface sediments fall into chronostratigraphic levels 4 and 5 as defined by the MARGO Project^22,21^. Level 4 classifies surface samples with age range of 0–4 cal ka BP where the chronologic control is based on stratigraphic constraints such as δ^18^O and %CaCO_3_. Level 5, in turn, describes surface samples that have no age control^21^. All North Atlantic surface samples with chronostratigraphic level 4 are located in the African continental margin while the rest of the basin (i.e., to the west of ca. 25°W), including the samples used to track the Atlantic ITCZ, fall into level 5. Due to the low sedimentation rate of ≤ 5 cm/kyr in the central tropical North Atlantic^27,22^, it is possible, in extreme cases, that these samples represent a period when the ITCZ occupied its northernmost position during the Holocene (*i.e.* the thermal maximum occurring from 10.5 to 5.4 kyr cal BP)^34^. Paleoclimatic records support a southward migration of the ITCZ over the late Holocene, as evidenced by dry conditions in northern South America inferred from %Ti and %Fe recorded in marine core ODP 1002 from Cariaco Basin^34^. Thus the ITCZ may have reached its modern position slightly south from those recorded by the faunal assemblage in the surface sediments, producing the offset between the R_N/Gg_ derived from surface samples and the modern mean annual position of the Atlantic ITCZ^7^. It is important to note that the lack of surface sediments in the region between 5° – 15°N and 50° – 70°W and a different seasonal succession of planktonic foraminifera species^28,29,35^ may also contribute to explain this small offset. For example, *N. incompta* generally reproduces in boreal spring and summer in the North Atlantic^28^ when the ITCZ reaches its northernmost position.

***The Holocene records from Tobago Basin***

We hypothesize that the low R_N/Gg_ values in the Tobago Basin core M35003-4 during the mid Holocene may be related to an ITCZ positioned to the north of the core site. The northward migration of the mean annual position of the ITCZ after the YD was probably fast, as suggested by the swift increase (decrease) in Ti (reflectance)^34,36^ from Cariaco Basin (Figs. 2c and S7a), which match well with an increase of our R_N/Gg_ record from core M35003-4 (Tobago Basin) just after the YD. However, both the Ti and the reflectance records from the Cariaco Basin indicate a more gradual southward migration of the ITCZ during the late Holocene^34,36^. Given the large uncertainties in the age model of core MS35003-4 after 5.66 cal ka BP (the uppermost AMS radiocarbon age from this core at 98cm; Table S1), we hypothesize that the uppermost samples from core M35003-4, which display high R_N/Gg_ values, may have recorded the gradual southward migration of the Atlantic ITCZ on the way to its modern mean annual location after 3 cal ka BP.

**References**

1. Peterson, R. G. & Stramma, L. Upper-level circulation in the South Atlantic Ocean. *Progress in Oceanography* **26,** 1–73 (1991).

2. Schott, F. A. *et al.* The Shallow and Deep Western Boundary Circulation of the South Atlantic at 5°–11°S. *Journal of Physical Oceanography* **35,** 2031–2053 (2005).

3. Zhang, D., McPhaden, M. J., Johns, W. E. & Al, Z. E. T. Observational Evidence for Flow between the Subtropical and Tropical Atlantic: The Atlantic Subtropical Cells*. *Journal of Physical Oceanography* **33,** 1783–1797 (2003).

4. Johns, W. E. *et al.* Annual Cycle and Variability of the North Brazil Current. *Journal of Physical Oceanography* **28,** 103–128 (1998).

5. Ischer, R. F., Schott, F. A., Fischer, J., Stramma, L. & Ischer, R. F. Transports and Pathways of the Upper-Layer Circulation in the Western Tropical Atlantic. *Journal of Physical Oceanography* **28,** 1904–1928 (1998).

6. Philander, S. G. H. *et al.* Why the ITCZ Is Mostly North of the Equator. *Journal of Climate* **9,** 2958–2972 (1996).

7. Schneider, T., Bischoff, T. & Haug, G. H. Migrations and dynamics of the Intertropical Convergence Zone. *Nature* **513,** 45–53 (2014).

8. Hastenrath, S. & Lamb, P. J. Climate dynamics of atmosphere and ocean in the equatorial zone: A synthesis. *International Journal of Climatology* **24,** 1601–1612 (2004).

9. Xie, S.-P. & Philander, S. G. H. A coupled ocean-atmosphere model of relevance to the ITCZ in the eastern Pacific. *Tellus A* **46,** 340–350 (1994).

10. Schott, F. A., Mccreary, J. P. & Johnson, G. C. *Shallow Overturning Circulations of the Tropical- Subtropical Oceans*. **147,** 261–304 (American Geophysical Union, 2004).

11. Roemmich, D. The Balance of Geostrophic and Ekman Transports in the Tropical Atlantic Ocean. *Journal of Physical Oceanography* **13,** 1534–1539 (1983).

12. Longhurst, A. Seasonal cooling and blooming in tropical oceans. *Deep Sea Research Part I: Oceanographic Research Papers* **40,** 2145–2165 (1993).

13. Locarnini, R. A. et al. World Ocean Atlas 2009. In: Levitus, S. (Ed.), temperature, NOAA Atlas NESDIS 68 vol. 1. U.S. Government Printing Office, Washington, D. C., 184 pp. (2010).

14. Antonov, J. I. et al. World Ocean Atlas 2009. In: Levitus, S. (Ed.), Salinity, NOAA Atlas NESDIS 69 vol. 2. U.S. Government Printing Office, Washington, D. C., 184 pp. (2010).

15. Garcia, H. E. et al. World Ocean Atlas 2009 In: Levitus, S. (Ed.), Dissolved Oxygen, Apparent Oxygen Utilization, and Oxygen Saturation, NOAA Atlas NESDIS 70 vol. 3. U.S. Government Printing Office, Washington, D. C., 344 pp. (2010).

16. Garcia, H. E. et al. World Ocean Atlas 2009 n: Levitus, S. (Ed.), Nutrients (phosphate, nitrate, and silicate), NOAA Atlas NESDIS 71 vol. 4. U.S. Government Printing Office, Washington, D. C., 398 pp. (2010).

17. De Boyer Montégut, C., Madec, G., Fischer, A. S., Lazar, A. & Iudicone, D. Mixed layer depth over the global ocean: An examination of profile data and a profile-based climatology. *Journal of Geophysical Research C: Oceans* **109,** 1–20 (2004).

18. Mignot, J., de Boyer Montégut, C., Lazar, A. & Cravatte, S. Control of salinity on the mixed layer depth in the world ocean: 2. Tropical areas. *Journal of Geophysical Research: Oceans* **112,** 1–12 (2007).

19. De Boyer Montégut, C., Mignot, J., Lazar, A. & Cravatte, S. Control of salinity on the mixed layer depth in the world ocean: 1. General description. *Journal of Geophysical Research* **112,** C06011 (2007).

20. Juggins, S. C2 user guide: Software for ecological and palaeoecological data analysis and visualization. (2003).

21. Kucera, M., Rosell-Melé, A., Schneider, R., Waelbroeck, C. & Weinelt, M. Multiproxy approach for the reconstruction of the glacial ocean surface (MARGO). *Quaternary Science Reviews* **24,** 813–819 (2005).

22. Kucera, M. *et al.* Reconstruction of sea-surface temperatures from assemblages of planktonic foraminifera: multi-technique approach based on geographically constrained calibration data sets and its application to glacial Atlantic and Pacific Oceans. *Quaternary Science Reviews* **24,** 951–998 (2005).

23. Telford, R. J., Li, C. & Kucera, M. Mismatch between the depth habitat of planktonic foraminifera and the calibration depth of SST transfer functions may bias reconstructions. *Climate of the Past* **9,** 859–870 (2013).

24. Ravelo, A. C., Fairbanks, R. G. & Philander, S. G. H. Reconstructing tropical Atlantic hydrography using planktontic foraminifera and an ocean model. *Paleoceanography* **5,** 409–431 (1990).

25. Schmuker, B. & Schiebel, R. Planktic foraminifers and hydrography of the eastern and northern Caribbean Sea. *Marine Micropaleontology* **46,** 387–403 (2002).

26. Steph, S., Regenberg, M., Tiedemann, R., Mulitza, S. & Nürnberg, D. Stable isotopes of planktonic foraminifera from tropical Atlantic/Caribbean core-tops: Implications for reconstructing upper ocean stratification. *Marine Micropaleontology* **71,** 1–19 (2009).

27. Cléroux, C., DeMenocal, P., Arbuszewski, J. & Linsley, B. Reconstructing the upper water column thermal structure in the Atlantic Ocean. *Paleoceanography* **28,** 503–516 (2013).

28. Chapman, M. R. Seasonal production patterns of planktonic foraminifera in the NE Atlantic Ocean: Implications for paleotemperature and hydrographic reconstructions. *Paleoceanography* **25,** PA1101 (2010).

29. Tedesco, K., Thunell, R., Astor, Y. & Muller-Karger, F. The oxygen isotope composition of planktonic foraminifera from the Cariaco Basin, Venezuela: Seasonal and interannual variations. *Marine Micropaleontology* **62,** 180–193 (2007).

30. Fairbanks, R. G., Wiebe, P. H. & Bé, A. W. H. Vertical Distribution and Isotopic Composition of Living Planktonic Foraminifera in the Western North Atlantic. *Science* **207,** 61–63 (1980).

31. Ortiz, J. D., Mix, A. C. & Collier, R. W. Environmental control of living symbiotic and asymbiotic foraminifera of the California Current. *Paleoceanography* **10,** 987–1009 (1995).

32. Gastrich, M. D. Ultrastructure of a new intracellular symbiotic alga found within planktonic foraminifera. *Journal of Phycology* **23,** 623–632 (2007).

33. Darling, K. F., Kucera, M., Kroon, D. & Wade, C. M. A resolution for the coiling direction paradox in Neogloboquadrina pachyderma. *Paleoceanography* **21,** PA2011 (2006).

34. Haug, G. H. Southward Migration of the Intertropical Convergence Zone Through the Holocene. *Science* **293,** 1304–1308 (2001).

35. Fairbanks, R. G. & Wiebe, P. H. Foraminifera and Chlorophyll Maximum: Vertical Distribution, Seasonal Succession, and Paleoceanographic Significance. *Science* **209,** 1524–1526 (1980).

36. Peterson, L. C., Haug, G. H., Hughen, K. A. & Röhl, U. Rapid changes in the hydrologic cycle of the tropical Atlantic during the last glacial. *Science (New York, N.Y.)* **290,** 1947–1951 (2000).

37. Ruhlemann, C., Mulitza, S., Muller, P. J., Wefer, G. & Zahn, R. Warming of the tropical Atlantic Ocean and slowdown of thermohaline circulation during the last deglaciation. *Nature* **402,** 511–514 (1999).

38. Hüls, M. & Zahn, R. Millennial-scale sea surface temperature variability in the western tropical North Atlantic from planktonic foraminiferal census counts. *Paleoceanography* **15,** 659–678 (2000).

39. Schlitzer, R. Ocean Data View. http://odv.awi.de (2016).

40. Zhang, Y. *et al.* Origin of increased terrigenous supply to the NE South American continental margin during Heinrich Stadial 1 and the Younger Dryas. *Earth and Planetary Science Letters* **432,** 493–500 (2015).

**Figure S1:**

**
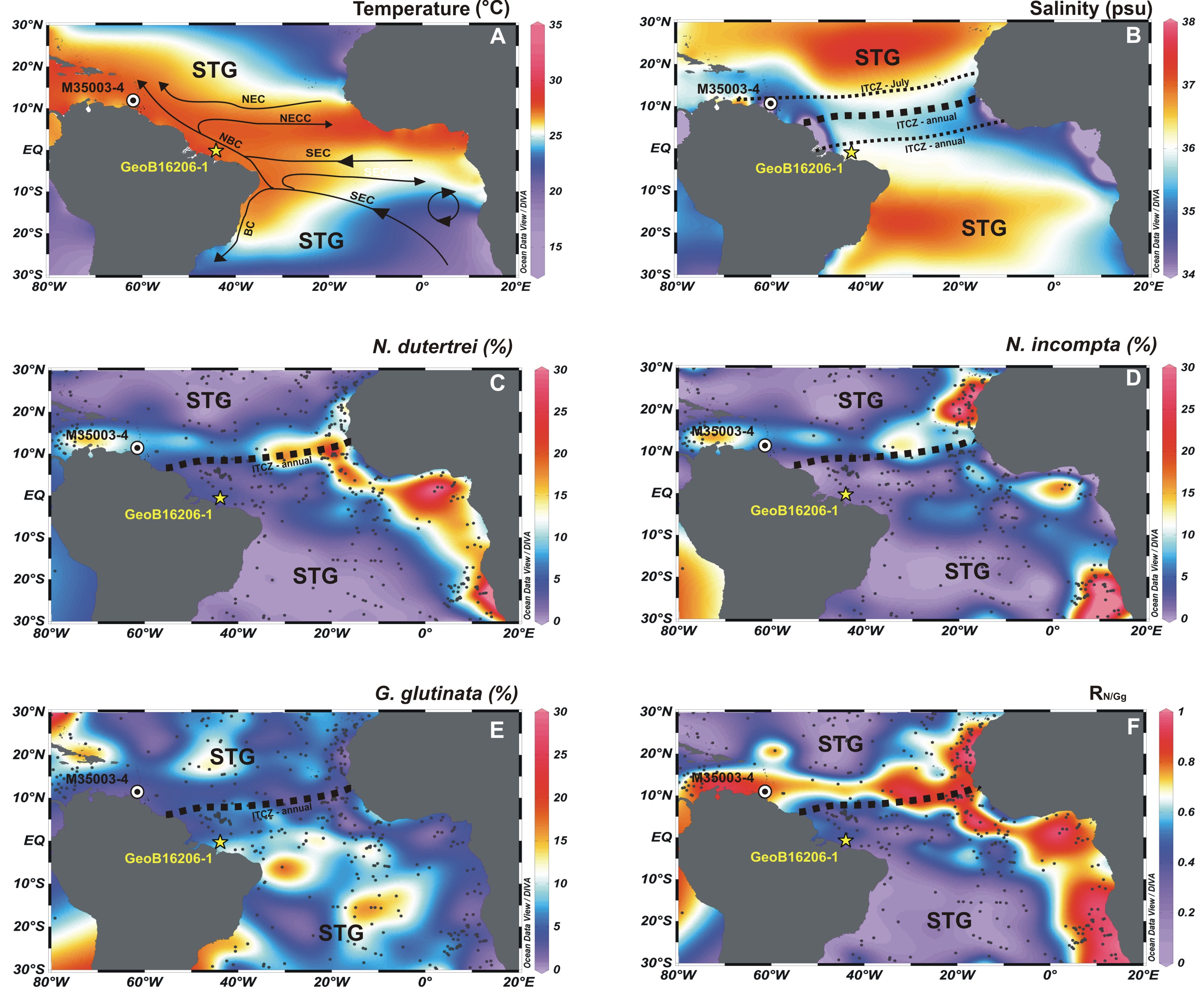
**

**Figure S2:**

**
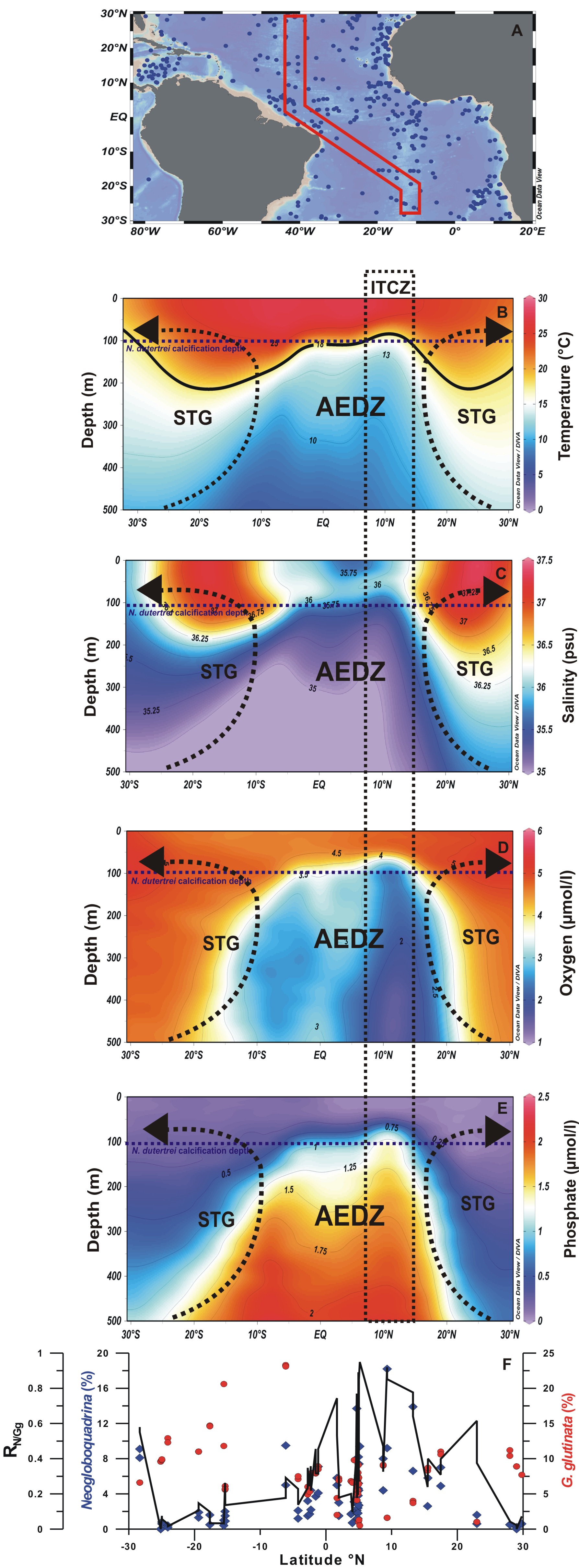
**

**Figure S3:**

**
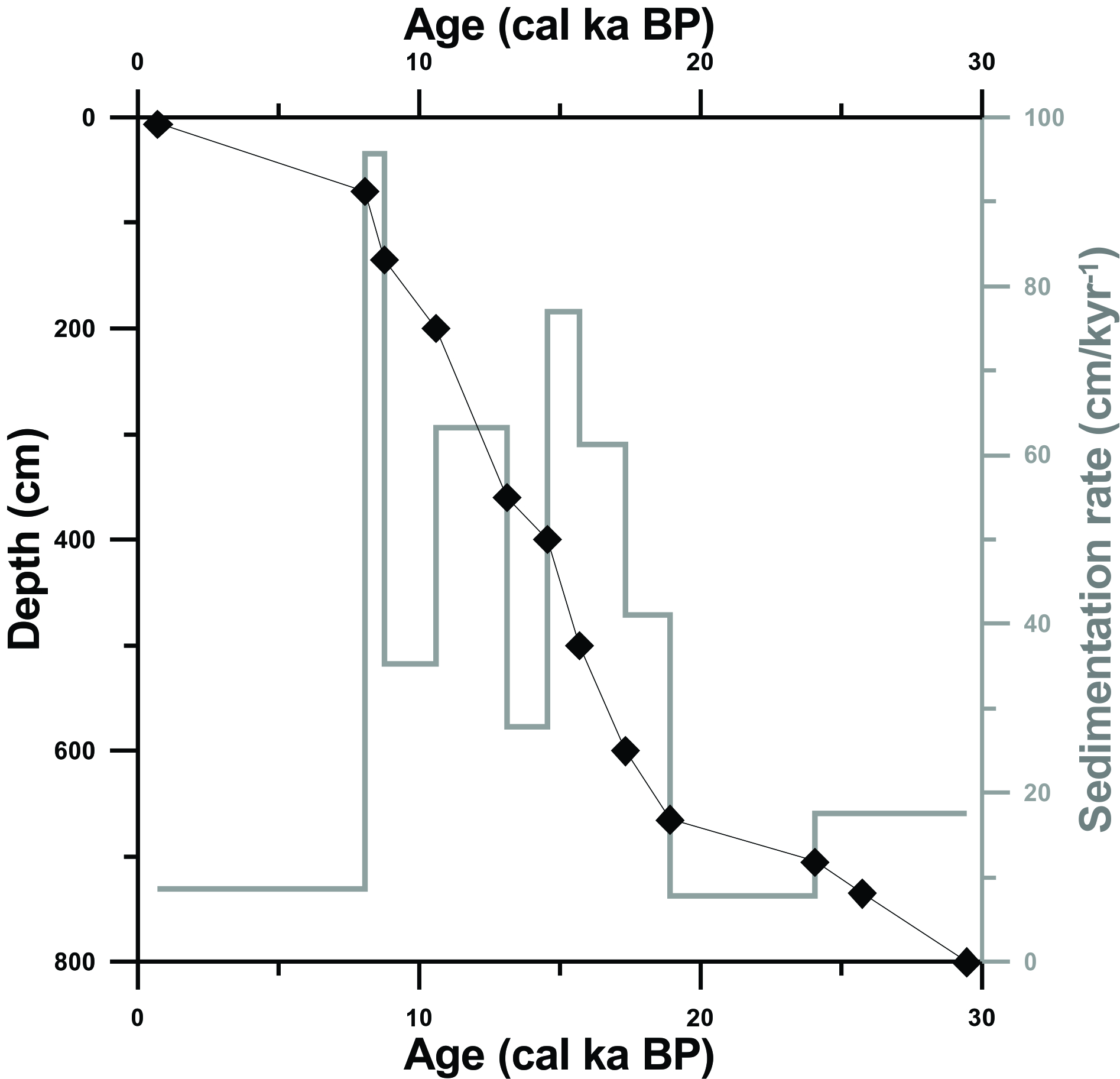
**

**Figure S4:**

**
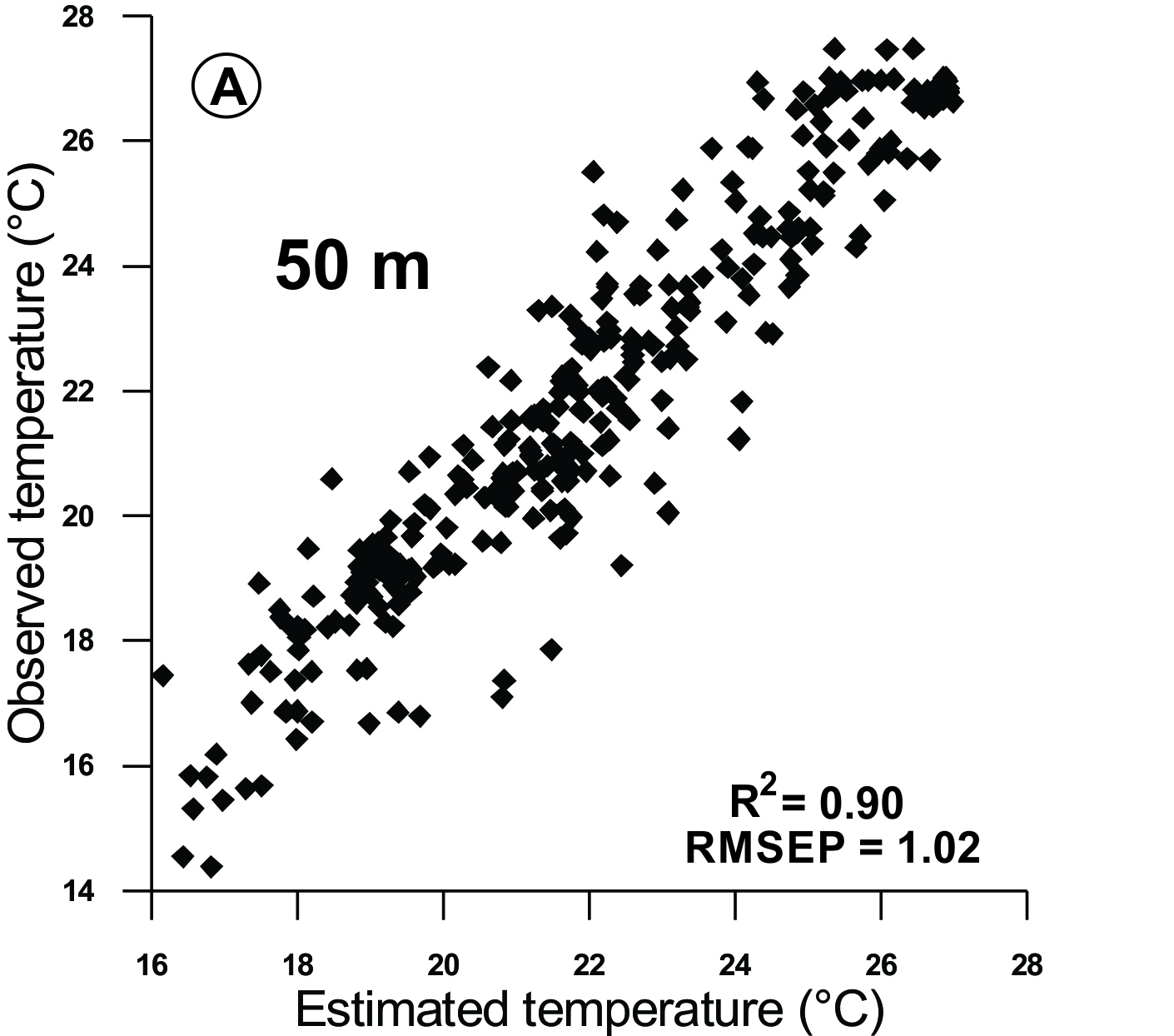
**

**Figure S5:**

**
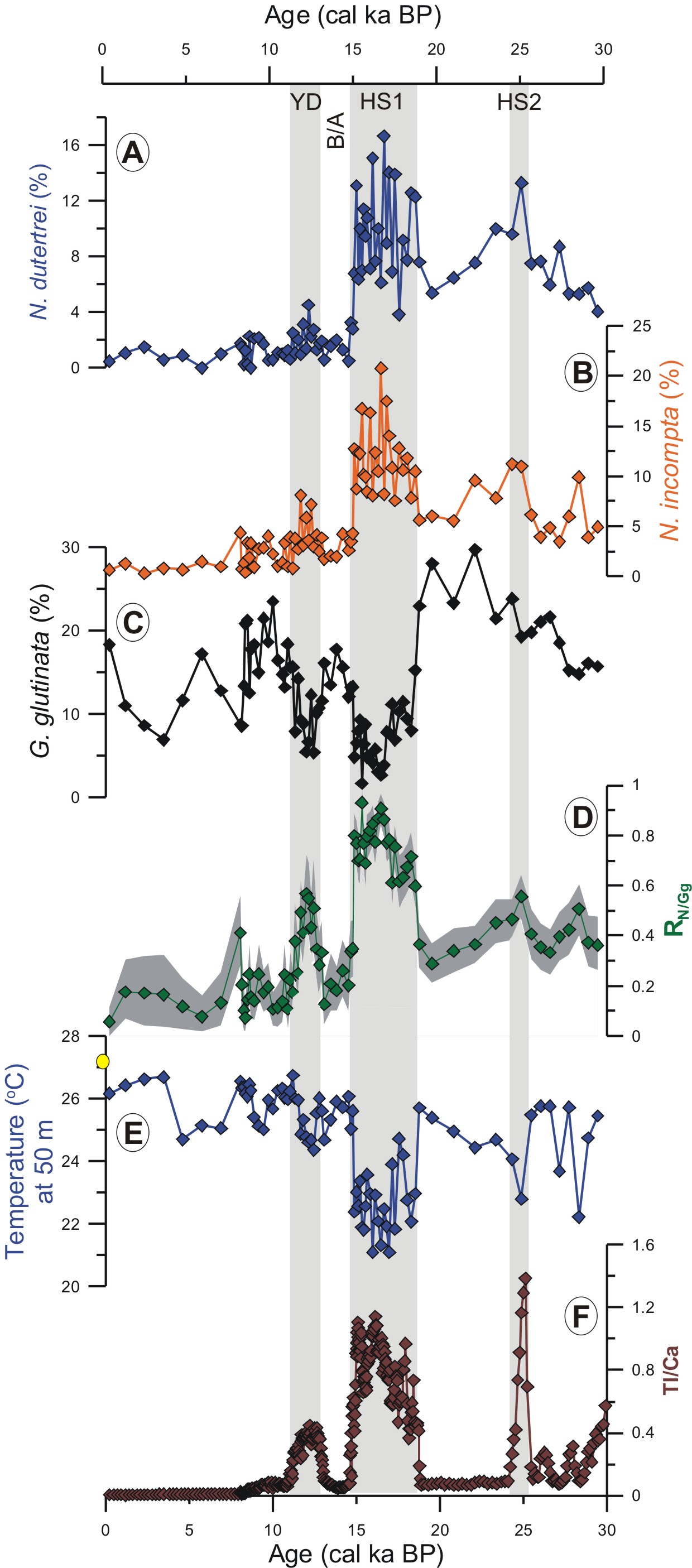
**

**Figure S6:**

**
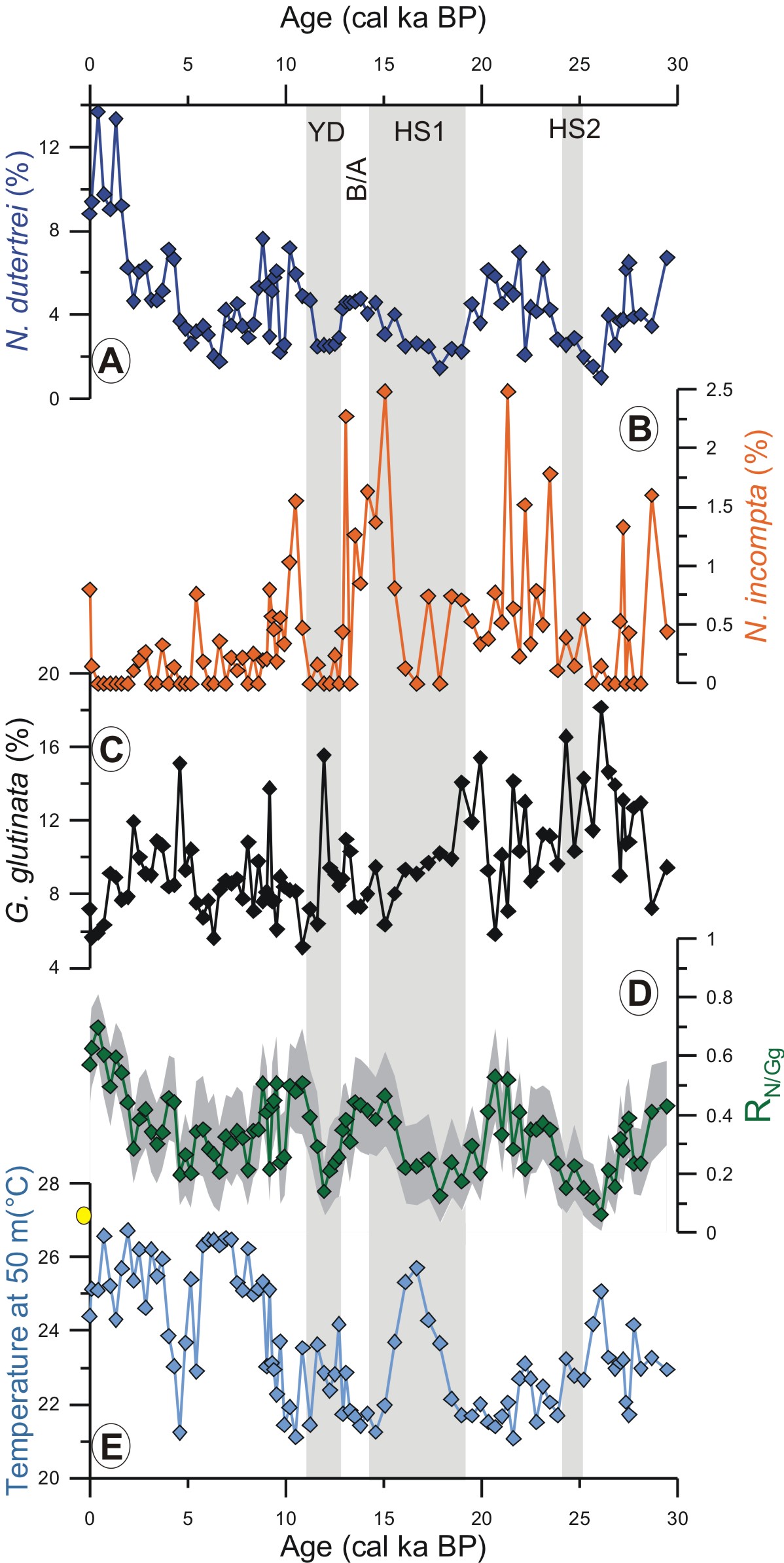
**

**Figure S7:**

**
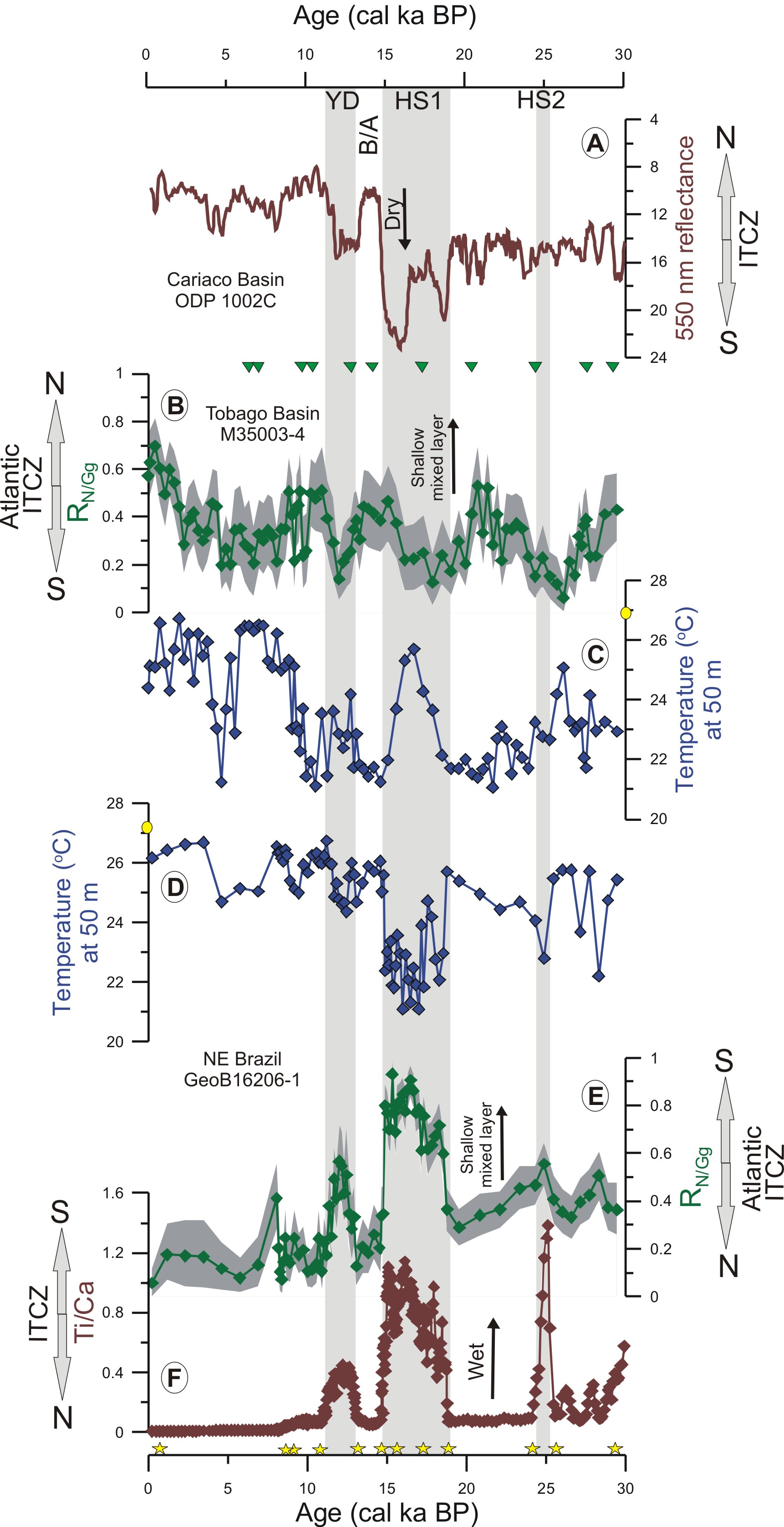
**

**Table S1**

| **Cores** | **Lab. No.** | **Core depth (cm)** | **Analysed material** | **Radiocarbon age**  **± 1σ error (yr BP)** | **Calibrated age**  **± 2σ error (cal ka BP)** | **References** |
| --- | --- | --- | --- | --- | --- | --- |
| GeoB16206-1 | Poz–50178 | 6 | *G. sacculifer* | 1180 ± 30 | 0.72 ± 0.065 | Zhang et al. (2015) |
| GeoB 6206-1 | Poz–52812 | 70 | *G. sacculifer* | 7610 ± 40 | 8.07 ± 0.095 | Zhang et al. (2015) |
| GeoB16206-1 | Poz–52740 | 135 | *G. sacculifer* | 8210 ± 50 | 8.75 ± 0.177 | Zhang et al. (2015) |
| GeoB16206-1 | Poz–50179 | 200 | *G. sacculifer* | 9690 ± 60 | 10.59 ± 0.165 | Zhang et al. (2015) |
| GeoB16206-1 | Poz–52741 | 360 | *G. sacculifer* | 11630 ± 50 | 13.12 ± 0.154 | Zhang et al. (2015) |
| GeoB16206-1 | Poz–50180 | 400 | *G. sacculifer* | 12840 ± 70 | 14.56 ± 0.397 | Zhang et al. (2015) |
| GeoB16206-1 | Poz–52742 | 500 | *G. sacculifer* | 13490 ± 70 | 15.69 ± 0.288 | Zhang et al. (2015) |
| GeoB16206-1 | Poz–50182 | 600 | *G. sacculifer* | 14630 ± 80 | 17.32 ± 0.249 | Zhang et al. (2015) |
| GeoB16206-1 | Poz–52744 | 665 | *G. sacculifer* | 16060 ± 80 | 18.90 ± 0.186 | Zhang et al. (2015) |
| GeoB16206-1 | Poz–52813 | 705 | *G. sacculifer* | 20390 ± 100 | 24.05 ± 0.282 | Zhang et al. (2015) |
| GeoB16206-1 | Poz–52814 | 735 | *G. sacculifer* | 21840 ± 100 | 25.76 ± 0.201 | Zhang et al. (2015) |
| GeoB16206-1 | Poz–52815 | 800 | *G. sacculifer* | 25790 ± 150 | 29.46 ± 0.454 | Zhang et al. (2015) |
| M35003-4 | KIA5085 | 98 | *G. ruber* (pink) | 5300 ± 50 | 5.66 ± 0.118 | Ruehlemann et al. (1999) |
| M35003-4 | KIA4223 | 110 | *G. ruber* (white) | 5920 ± 40 | 6.33 ± 0.085 | Ruehlemann et al. (1999) |
| M35003-4 | KIA5084 | 163 | Mixed* | 8480 ± 60 | 9.10 ± 0.155 | Ruehlemann et al. (1999) |
| M35003-4 | KIA4224 | 190 | *G. ruber* (white) | 9150 ± 50 | 9.93 ± 0.199 | Ruehlemann et al. (1999) |
| M35003-4 | KIA4225 | 225 | *G. ruber* (white) | 10800 ± 90 | 12.27 ± 0.298 | Ruehlemann et al. (1999) |
| M35003-4 | KIA4226 | 257 | *G. ruber* (white) | 12220 ± 70 | 13.68 ± 0.190 | Ruehlemann et al. (1999) |
| M35003-4 | KIA4227 | 290 | *G. ruber* (white) | 14210 ± 90 | 16.70 ± 0.321 | Ruehlemann et al. (1999) |
| M35003-4 | KIA4228 | 310 | *G. ruber* (white) | 16140 ± 100 | 18.99 ± 0.238 | Ruehlemann et al. (1999) |
| M35003-4 | KIA6973 | 380 | *G. ruber* (white) | 20260 ± 110 | 23.90 ± 0.306 | Hüls and Zahn (2000) |
| M35003-4 | KIA4229 | 420 | *G. ruber* (white) | 23230 ± 210 | 27.15 ± 0.467 | Ruehlemann et al. (1999) |
| M35003-4 | KIA4230 | 450 | *G. ruber* (white) | 25060 ± 260 | 28.69 ± 0.606 | Ruehlemann et al. (1999) |

*Mixed planktonic foraminifera

**Supplementary Figure Captions**

**Figure S1:** Regional setting, location of cores and modern distribution of planktonic foraminifera between 30°N and 30°S in the Atlantic Ocean. (**a**) Mean annual surface temperature^13^ and schematic surface circulation in the tropical Atlantic Ocean^1^ showing the location of the cores GeoB16206–1 (this study) and M35003–4^37,38^. (**b**) Mean annual surface salinity^14^ and modern location of the atmospheric Intertropical Convergence Zone (ITCZ; thick dashed line) and its seasonal location during January and July (thin dashed lines)^6,7^. (**c–f**) Modern distribution of planktonic foraminifera species from 407 surface sediment (black dots; MARGO Project^22^); (**c**) *Neogloboquadrina dutertrei*, (**d**) *Neogloboquadrina incompta*, (**e**) *Globigerina glutinata,* and (**f**) %*Neogloboquadrina* / (%*Neogloboquadrina* + %*G. glutinata*) (R_N/Gg_) ratio. The mean surface currents in (**a**) are: Brazil Current (BC), North Brazil Current (NBC), North Equatorial Counter Current (NECC), North Equatorial Current (NEC) and South Equatorial Current (SEC), STG: subtropical gyre. Figure created using Ocean Data View software^39^ (ODV - version, 4.7.4., http://[odv.awi.de](http://odv.awi.de/), 2015).

**Figure S2:** Latitudinal cross–section of the upper 500 m in the western Atlantic Ocean between 30°N–30°S. (**a**) Location of the 407 surface sediment samples (blue dots) in the tropical Atlantic from the MARGO Project^22,21^. The red polygon depicts the latitudinal transect used in panels **b–f**. Annual mean (**b**) temperature^13^, (**c**) salinity^14^, (**d**), oxygen concentration^15^ and (**e**) phosphate concentration^16^. Black dashed arrows represent the large equatorial upwelling between 10°N–10°S referred to as the Atlantic Equatorial Divergence Zone (AEDZ). The thick black line in (**b**) highlights the 18°C isotherm generally used to define the depth of the thermocline. Horizontal blue dashed line indicates the apparent calcification depth (~100 m water depth) of *Neogloboquadrina dutertrei* in the tropical Atlantic Ocean^26,27^. (**f**) abundance of *Neogloboquadrina* (grouped *N. dutertrei* and *Neogloboquadrina incompta*) and *Globigerina glutinata* as well as the ratio between them R_N/Gg_ (%*Neogloboquadrina* / (%*Neogloboquadrina* + %*G. glutinata*)). The vertical dashed box indicates the location of the annual range of the Intertropical Convergence Zone (ITCZ) and its associated shallower mixed-layer between 5°–15°N, the oceanic counterpart of the ITCZ, the Atlantic ITCZ. Figure created using Ocean Data View software^39^ (ODV - version, 4.7.4., http://[odv.awi.de](http://odv.awi.de/), 2015).

**Figure S3:** Age model (black line) and sedimentation rate (grey line) for core GeoB16206-1 (ref.^40^). Black diamonds indicate calibrated AMS ^14^C ages.

**Figure S4:** Calibration curve between observed and estimated temperature for the upper water column using surface sediments from the tropical Atlantic Ocean between 30°N and 30°S from the MARGO database^22,21^ and applying the Modern Analogue Technique (MAT) at 50 m water depth.

**Figure S5:** Records for core GeoB16206–1 collected off NE Brazil. (**a–c**) Downcore abundance of planktonic foraminifera species (**a**) *Neogloboquadrina dutertrei*, (**b**) *Neogloboquadrina incompta,* and (**c**) *Globigerina glutinata.* (**d**) R_N/Gg_ ratio (%*Neogloboquadrina* / (%*Neogloboquadrina* + %*G. glutinata*)). (**e**) Reconstructed subsurface temperature at 50 m derived from planktonic foraminifera Modern Analog Technique. (**f**) Ti/Ca ratio as a proxy for rainfall regime over NE Brazil. Vertical gray bars indicate Heinrich Stadials 2 and 1 (HS2–1), and the Younger Dryas (YD). B/A: Bølling–Allerød. Grey shading in (**d**) indicates the 95% confidence interval. Yellow circle in (**e**) indicate modern temperatures at 50 m off NE Brazil.

**Figure S6:** Records for core M35003-4 collected at the Tobago Basin^38^. (**a–c**) Downcore abundance of planktonic foraminifera species (**a**) *Neogloboquadrina dutertrei*, (**b**) *Neogloboquadrina incompta,* and (**c**) *Globigerina glutinata.* (**d**) R_N/Gg_ ratio (%*Neogloboquadrina* / (%*Neogloboquadrina* + %*G. glutinata*)). (**e**) Reconstructed subsurface temperature at 50 m derived from planktonic foraminifera Modern Analog Technique. Vertical gray bars indicate Heinrich Stadials 2 and 1 (HS2–1), and the Younger Dryas (YD). B/A: Bølling–Allerød. Grey shading in (**d**) indicates the 95% confidence interval. Yellow circle in (**e**) indicate modern temperatures at 50 m at the Tobago Basin.

**Figure S7:** Antiphase relationship between NE Brazil and Tobago/Cariaco Basin over the last 30 cal ka BP. (**a**) Cariaco Basin core ODP 1002C reflectance showing wet/dry conditions linked to meridional shifts of the Intertropical Convergence Zone (ITCZ)^36^. %*Neogloboquadrina* / (%*Neogloboquadrina* + %*G. glutinata*) (R_N/Gg_) ratio as a proxy for the Atlantic ITCZ in the (**b**) Tobago Basin core M35003-4 and the (**e**) NE Brazil core GeoB16206-1. Subsurface temperature at 50 m derived from Modern Analog Technique in the (**c**) Tobago Basin and (**d**) off NE Brazil. (**f**) Ti/Ca ratio as a proxy for precipitation over NE Brazil. Vertical gray bars indicate Heinrich Stadials 2 and 1 (HS2–1) and the Younger Dryas (YD). The Bølling–Allerød (B/A) is also indicated. Calibrated AMS ^14^C ages are shown as green triangles for core M35003-4^37,38^ and yellow stars for core GeoB16206-1^40^. Grey shading in (**b**) and (**e**) indicates the 95% confidence interval. Yellow circles in (**c**) and (**d**) indicate modern temperatures at 50 m at both core locations^13^. Note the inverted axis in (**a**).

**Table S1:** Raw AMS ^14^C data and calibrated ages of sediment cores GeoB16206–1 (ref.^40^) and M35003-4 (ref.^37,38^).
